# Challenges and prospects for implementation of community health volunteers’ digital health solutions in Kenya: a qualitative study

**DOI:** 10.1186/s12913-020-05711-7

**Published:** 2020-09-21

**Authors:** Pauline Bakibinga, Eva Kamande, Lyagamula Kisia, Milka Omuya, Dennis J. Matanda, Catherine Kyobutungi

**Affiliations:** 1 African Population & Health Research Center, Manga Close, Off Kirawa Road, P.O. Box 10787-00100, Nairobi, Kenya; 2 Population Council, Avenue 5, Rose Avenue, P.O. Box 17643-00500, Nairobi, Kenya

**Keywords:** Digital health, Challenges, Prospects, Implementation, Kenya

## Abstract

**Background:**

The value of digital health technologies in delivering vital health care interventions, especially in low resource settings is increasingly appreciated. We co-developed and tested a decision support mobile health (m-Health) application (app);with some of the forms used by Community Health Volunteers (CHVs) in Kenya to collect data and connected to health facilities. This paper explores the experiences of CHVs, health workers and members of Sub-County Health Management Teams following implementation of the project.

**Methods:**

Data were collected in December 2017 through in-depth interviews and focus group discussions with CHVs and key informant interviews with health care workers and health managers in Kamukunji sub-County of Nairobi, Kenya. Data coding and analysis was performed in NVivo 12.

**Results:**

Regarding users and health managers’ perceptions towards the system; three main themes were identified: 1) variations in use, 2) barriers to use and 3) recommendations to improve use. Health workers at the private facility and some CHVs used the system more than health workers at the public facilities. Four sub-themes under barriers to use were socio-political environment, attitudes and behaviour, issues related to the system and poor infrastructure. A prolonged health workers’ strike, the contentious presidential election in the year of implementation, interrupted electricity supply and lack of basic electric fixtures were major barriers to use. Suggestions to improve usage were: 1) integration of the system with others in use and making it available on users’ regular phones, and 2) explore sustainable motivation models to users as well as performance based remuneration.

**Conclusions:**

The findings reveal the importance of considering the readiness of information and communication technologies (ICT) users before rollout of ICT solutions. The political and sociocultural environment in which the innovation is to be implemented and integration of new solutions into existing ones is critical for success. As more healthcare delivery models are developed, harnessing the potential of digital technologies, strengthening health systems is critical as this provides the backbone on which such innovations draw support.

## Background

There is growing evidence of the value of digital technologies in promoting access to healthcare. The World Health Organisation (WHO) defines digital health as ‘a discrete functionality of digital technology that is applied to achieve health objectives’ [1]. The growing benefits of digital health are evident in patient management, research, and support to low cadre health workers, including community health volunteers, data collection and analysis, disease surveillance, among other uses. This is particularly important in low resource settings [2]. The value of digital technologies is appreciated for its ability to transcend geographical barriers while allowing real-time access to vital health services [3, 4].

There are national, regional and global guidelines and strategies towards utilisation of digital health to promote the achievement of universal health coverage. The WHO recently released the first set of guidelines [5], to this end, noting that digital health cannot replace non-functional health systems. Electronic health (e-health) readiness, is defined as ‘preparedness of healthcare institutions or communities for the anticipated change brought by programs related to information and communications technology’ [6]. Without e-readiness, implementation of programs is challenging. The current East African Community (EAC) Health Sector Investment Priority Framework (2018–2028) [7], in all the nine priority areas, stresses the regional block’s commitment to harness the potential of e-health on the road to universal health coverage. The EAC is composed of six partner states: Burundi, Kenya, Rwanda, United Republic of Tanzania, South Sudan and Uganda. The EAC health secretariat, through the ministers’ of health has called on countries to implement e-health strategies, with a recommendation that the East African Science and Technology Commission conduct a regional e- health readiness assessment [8].

Kenya launched its first National e-health Strategy in 2011(2011–2017) [9] with a rallying call to strengthen the health system and subsequently extend equity in health care to the poor and marginalized population. Five key areas were identified: telemedicine; electronic health records (health information systems); information for citizens; m-health (mobile technologies in health); and eLearning or distance education for health professionals. The strategy was followed by the National e-Health Policy (2016–2030) reiterating the creation of an enabling environment that promotes adoption, implementation and use of e-Health at all levels of service delivery. The e- Health strategy and policy were supported by the Kenya Health Policy (2014–2030) that in one of its objectives aims to “plan, design and install ICT infrastructure and software for the management and delivery of essential healthcare”. Although Kenya, and a few other countries in the EAC have implemented their national e-health strategies, lack of scientific evidence on the benefits of e-health interventions, how they work under different conditions within health systems remain major limitations to evidence-based policy and programming.

Kenya, is a leading economic and technology hub in the East African Community, contributing to 40% of the region’s Gross Domestic Product [10]. The country has one of the highest mobile phone penetration rates, worldwide [11]. Kenya’s growth in ICT has enabled the implementation of various digital innovations for the wellbeing of Kenyans. Such include mobile money--a vital financial solution to Kenyans across socio-economic groups. The health system has attempted to unlock the potential of technology in health. Numerous e-health innovations have been developed and implemented in the country but very few, if any, have gone to scale due to numerous challenges [11, 12]. There is need to identify and address the gaps in order to have clear directions on where to focus investment, in a coordinated manner. With limited research on technological innovations, the “quiet revolution” being experienced in Kenya faces the risk of wasted resources and potential creation of a digital health divide in health care.

Lately, Kenya has started to include ICT in national policy and planning for human resources for health. A broad range of legislation, regulations, and guidelines now exist that define the requirements of employment, and development of the health workforce as regards ICT. For instance, the National eHealth policy (2016–2030) recognizes the importance of ICT training and capacity building for health care workers as one of the key policy orientations. This policy orientation stipulates the integration of ICT into existing education and training at different levels; continuous education, sensitization and technical support to eHealth users. It also promotes continuous professional development (CPD) through e-learning [13]. Further, the scheme of service stipulates a certificate in computer application skills from a recognized institution as one of the minimum employment qualification for community health service personnel such as community health assistants and health care worker like clinical officers and nursing personnel [14–16]. To enforce this, National Continuing Professional Development Regulatory Framework, which provides a harmonized mechanism to address on-going professional development for Kenyan health care workers, outlines ICT as among other cross cadre CPD course that shall account to 10% of the required CPD points This provides healthcare workers who many not have had the opportunity to attain ICT knowledge and skill during the preservice education a platform to do so in relation to their profession duties.

Several digital pilot projects, involving low cadre health workers such as community health volunteers (CHVs) have demonstrated improvement in health service access and utilisation as a result of interventions focussing on CHVs. Within the WHO African region, the majority of implementation projects have mostly shown improvements in maternal and child health care as a result of better service delivery by CHVs, [17, 18]. In spite of the many pilot digital health projects implemented in SSA and in Kenya in particular, there is limited data on lessons learned especially in urban poor settings.

Building on lessons learned across SSA and with the aim of strengthen community health information and referral system at the community level in Kenya. We therefore developed an innovative digital application [19] that was also in response to a specific call for innovations to improve maternal and newborn wellbeing and survival in six counties identified as having the worst maternal and newborn health indicators; Bungoma, Garissa, Homa Bay, Kakamega, Turkana and Nairobi City [20]. Within Nairobi, informal settlements (slums) were identified as an area of focus given that they, compared to other areas of the city, have the very high maternal, newborn and child mortality rates [21]. The work was further informed by the government’s call to harness the role of digital technologies in health, as highlighted above [22]. As reported elsewhere [19], the mobile phone and web-application system we developed communicate over the internet to link the CHVs and health facilities. The mobile application used by the CHVs was a data capture module designed to replace selected paper based Ministry of Health (MoH) reporting tools. The functionality of the system was enhanced by the integration of a decision support function to enable identification of high risk cases and better management of community referrals. This was to further allow integration of patient data from the community to the health facility and enhanced better case management of referrals. Data quality was improved by validation checks that limited the submission of incomplete reports. The data from the community was accessed in real-time through the web by the health care providers and community health assistants (CHAs). Throughout the system development and implementation, CHVs views were included. The aim of this paper is to explore the experiences of CHVs, health workers and members of Sub-County Health Management Teams following implementation of the project in a bid to highlight challenges and opportunities presented by such processes in this and similar settings.

### Theory of Change

**Fig. 1:**
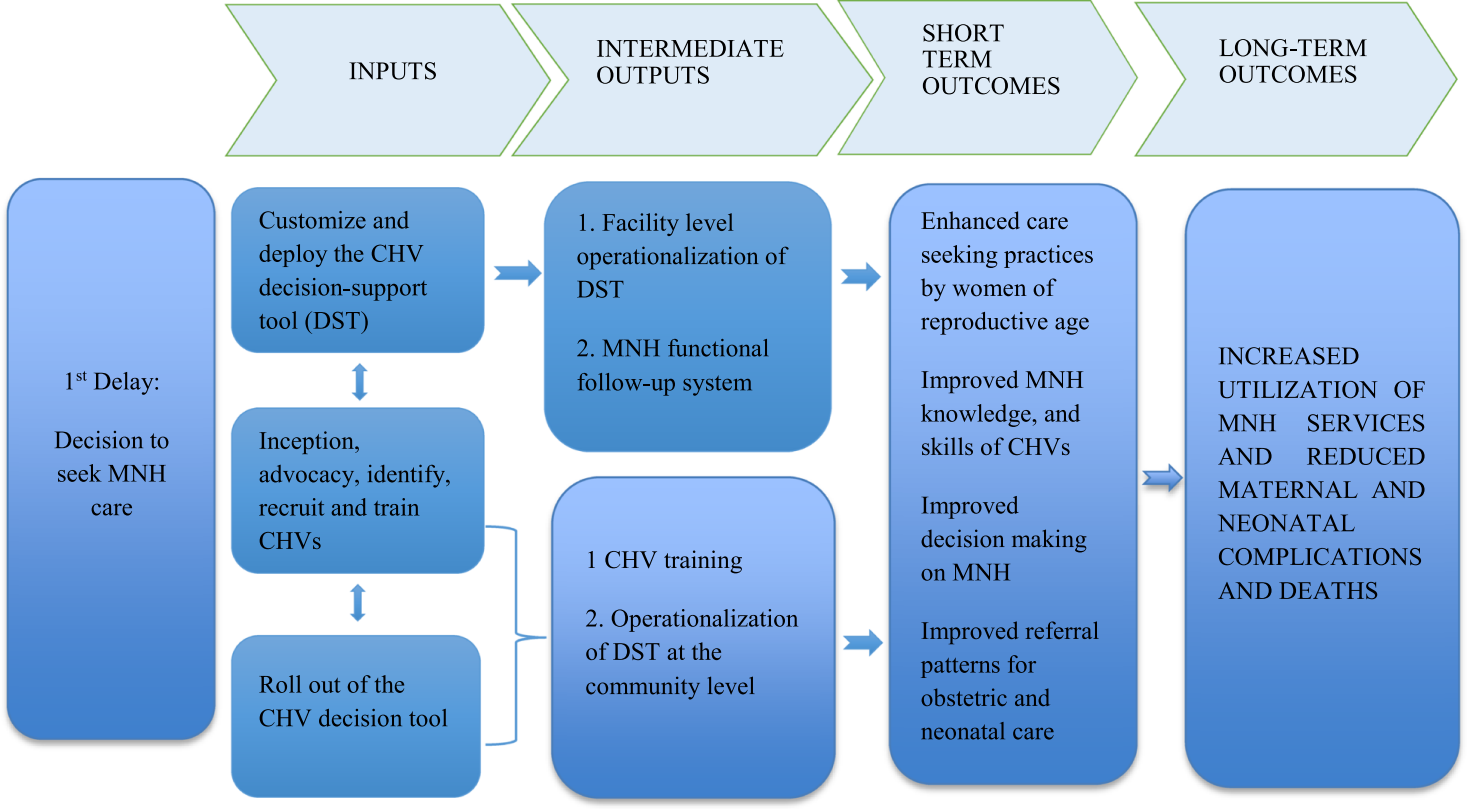
Intervention Theory of Change

As described elsewhere and demonstrated in Fig. 1, our theory of change was based on the assumption that a digitalized decision- support module of the application would enable the CHVs identify pregnant women, new mothers and their newborns exhibiting danger signs hence enable correct and timely decisions on referral for care in health facilities [19]. In essence, CHVs with more knowledge and skills on the needs of women and neonates at risk would be in a better position to care for community members in need of healthcare. As such, there would be an increase in the utilisation of mother and newborn services and a decrease in morbidity and mortality in the urban slums.

The inputs and processes described in Fig. 1 delivered the short and long term outcomes in addressing the delay in seeking maternal, newborn health (MNH) services. The digital application (m-PAMANECH) was developed to use a mobile and web-portal digital solutions that link the demand and supply of maternal and newborn services. The application had the official MoH 513 (household register), MoH 514 (service delivery log book) and MoH 100 (referral tool) used by the CHVs and the MoH 515 (CHA Summary) used by the CHAs. To improve the functionality and the utility of m-PAMANECH, a decision support tool (DST) was integrated in the application. The DST assisted the CHVs screen danger signs among pregnant women, newborns (0-28 days) and mothers in the immediate postpartum (within 6 weeks post birth). Consequently identifying cases that require immediate intervention by timely and correct referring to the health facility. Following customization of the application, m-PAMANECH was operationalized at the community and the five health centres providing MNCH services, by the recruitment and training of CHVs and health care workers. At the community level, a dedicated team of 50 trained CHVs accessed the m-PAMANECH application on the mobile handsets.

With the different access levels created for every user, the CHAs and the facilities accessed the submitted data remotely through the web. Allowing clinicians to treat referred patients and record their treatments and the sub-county community health strategy focal person and the CHAs, to follow-up on the CHVs through the system. The use of the application by the CHVs and the follow up by the CHAs ensured the intervention was delivered as expected, support was sustained and non- adherence to the intervention captured and documented. The effects of the inputs and immediate outcomes were assessed by examining the results in the population that the application was intended to serve. These results include increased level of MNH knowledge and expertise of the CHVs, timely care seeking decisions and enhanced care seeking behaviours ultimately increasing the utilization of MNH services, reduced complications and deaths.

## Methods

### Study setting and design

This study is based on a quasi-experimental study that was implemented in Kamukunji and Embakasi sub-Counties in Nairobi city [19]. This study focuses on qualitative data collected in Kamukunji sub-County which served as the intervention site. Kamukunji sub-county covers informal settlements, which, like other slums, are characterized by high level of poverty, limited health and social amenities, and adverse health outcomes. The project worked with seven community units (CUs), five health facilities and a dedicated team of 50 CHVs served as the intervention group in Kamukunji. The CUs in Kamukunji were Airbase, Eastleigh south, Kosovo, Sagana, Vihiga, Majengo and Okoa Maisha.

Following 1 year of implementation, qualitative assessments of CHVs’ and CHAs’ current work experiences with the digital application were conducted in Kamukunji. The assessment covered usability, barriers and opportunities for improvement. The participants were purposively selected and interviews administered through face-to-face interactions. In total, 35 participants were approached. All approached participants gave consent and completed the interview. None withdrew consent or dropped out. Table 1, presents a summary of all participants involved in this study.

**Table 1:** Group Composition and Discussion Guide for the Qualitative Interviews

Characteristics	Participants (***N*** = 35)	
	CHVs(***n*** = 25)	Health providers^**a**^ and sCHMTs (***n*** = 10)
Male	3	4
Female	22	6
**Age in years**		
20–30	4	4
> 30–40	8	2
> 40–50	10	1
+	3	1
**Education status**		
Pre- primary and primary	13	
Secondary	9	
Post-secondary	3	8
**Issues discussed**	Data collection experiences (electronic versus manual) and role in the provision of community health services	Access to and quality of and data collection experiences (electronic versus manual)

^a^Age and education status of two are missing

### Study population

The CUs were purposively selected. The selection was guided by the sub-county health management team based on the CUs with poor maternal and child indicators and hence would benefit from the intervention [19]. The project pre-requisite requirements were all CHVs to have undergone basic community health strategy, and MNCH technical training; the latter includes danger signs during pregnancy, new-born and postpartum period. Only the CHVs in intervention site received a refresher training on danger signs, basic operation of smartphone, and an intensive training on mobile reporting.

The health care workers were selected by the sub-county health management teams and were either clinical officers or nurses stationed at critical points in the facility i.e. outpatient or maternity departments. A majority of the health care workers were civil servants and therefore should have met the minimum requirements of appointment as outlined in the scheme of service for these personnel [14–16] . One of the minimum requirement being a certificate in computer application skills from a recognised institution. The health care workers from the intervention site were trained on how to navigate the internet and the web-application system including the integration of the mobile and web-application interface. An operation manual was also provided to users. All users, at the community and health facility, received frequent on-job training and mentorship sessions. To ensure sustainable on-going training, the CHAs were trained as trainers of trainers.

Health care workers in the selected health facilities and members of the sub-County Health Management teams (sCHMTs) were purposively selected to participate in this study. Some respondents were selected due to their role in the project. Respondents in the focus group discussions (FGDs) and in-depth interviews (IDI) were approached face to face while sCHMTs and health provider who participated in the key informant interviews were approached by telephone. All interviews were conducted face to face in December 2017.

### Research team

Three of five of the authors (PB, EK, LK, MO, DM and CK) were involved in the data collection process, including testing the tools, conducting quality control checks and facilitating interview sessions. All the authors are experienced researchers with interests in health and health systems. Additionally, the team engaged experienced research assistants who were graduates in relevant fields including Public Health, had experience in qualitative research, experience working in the slums, and fluency in English and Swahili. A 1 week intensive training on the project objectives, study guides, informed consent and interviewing techniques was conducted. This was further complimented with demonstration of interview techniques, role-plays and group discussions and field logistics.

### Data collection

The qualitative interview guides were developed in English and translated to Swahili. Back translation was done to ensure quality and accuracy when compared to the English version. Piloting of the instruments was conducted in Shauri Moyo in Kamukunji sub-county to ensure trainees grasped the questions and that the data collection tools captured as expected. The guides were revised based on outcomes of the pilot study (Additional file 1). Before commencement of the study, the data collectors were not known to the respondents. All the participants were brief on the objective of the study. The data were collected through focus group discussions (FGDs) with purposively selected CHVs and women who met the inclusion criteria, whereas in-depth interviews (IDIs) were only conducted with selected CHVs. FGDs and IDIs were conducted by a moderator in Swahili, assisted by a note taker. Key informant interviews (KIIs) were conducted in English with health care workers, CHAs and sub-County health management teams. The discussion notes were supplemented by observational notes for each interview. The notes also captured non-verbal ques. Each interview took approximately 45 to 60 min. The interviews were audio recorded and held in privacy, in spaces that were free of attentive eyes, eavesdroppers, threat of sanctions, and pressure from non-participants. FGDs were conducted in community hall and churches during the weekdays and school halls during the weekdays. KIIs were conducted in respondents’ offices while IDIs were being carried out at the respondents’ residence. A total of 5 IDIs, 10 KIIs, and 6 FGDs were conducted.

### Analysis

In order to describe and interpret the participants’ experiences, views, and perceptions, audio recorded discussions conducted in Kiswahili were translated to English and transcribed by an experienced transcriber into word files. The word files were uploaded onto NVivo 12 for coding and analysis. The transcribed discussions and interviews were coded using a preliminary coding scheme. The scheme was informed by the themes pre-identified in the qualitative study guides. The research team discussed and agreed on the coding frame. This informed assignment of the different data sections to categories. Thereafter content analysis was conducted. PB coded the transcripts using NVivo 12 software. EK, MK, DM, LK and PB reviewed the coded data to identify patterns and connections to themes. DM and PB led the interpretation process.

Findings from the CHVs were triangulated with in-depth interviews data from health workers and key informant interviews from the community strategy coordinator and CHAs. The discussion notes were further supported by observational notes made during field visits.

## Results

In assessing the usability of the system, three main themes 1) variations in use, 2) barriers to use, and 3) recommendations to improve use, were identified (Table 2).

**Table 2:** User perspectives on implementing the CHV DST

Theme	Categories	Codes
Variation in use	Users’ experiences	Positive experiences/benefits of using the system
		Negative experiences in using the system
	Limited users’ experiences	Type of facility
		CHV attributes
Barriers to use	Socio-political environment	Infrastructure available
		Health workers’ strike
		Electioneering period
	Attitudes and behaviour	Expectations
		Lack of knowledge and skills-ICT
	Issues related to the system	Network coverage
		Nature of the gadgets
		Nature of the system
Recommendations to improve the system	Suggestions for enhancing usage	Integrate the system with others
		Provide extra motivation for users, including performance based remuneration
		Provide basic ICT skills for users
		Strengthen ICT infrastructure

### Variation in use

The private facility and some CHVs used the system more than health workers at the public facilities. Generally, the system was well received by those who used it and appreciated for its benefit.

- Users’ experiences’.
  i. Positive experiences and or benefits of using the system.

The system was appreciated as beneficial in improving work-life experiences as highlighted below:

*“The phone is very much okay and it is also very easy than carrying papers……it prevents you from exposing yourself with book. Now the phone has brought….it has become technology we say technology. When you come to someone’s place like this, 2 min, I have a phone, you recognize us in the community. What we do now we are using the phone,’ and it makes the work easier*” IDI CHV.

*“…. it’s very friendly it’s been okay eh…uhm…I’m lacking words to put this, the user friendly of the mobile phone. It’s so reliable by the way and it is like a backup method yeah, information doesn’t get lost any how and a CHV will not tell you ‘the book is lost or I forgot the book.’ They have the phone with them they can report anytime, uh…uh-huh they can report any time….the phone is portable…it’s so friendly”* KII CHA.

*“It makes my work easier because even at night I can just look at my data and view them, it is not a must I go and write them in the office or following them in the community, I can just check through my mobile phone and see what they are doing, yeah.”* KII CHA.

ii. Negative experiences in using the system.

On the other hand the processes surrounding use were a deterrent to effect use as exemplified below:

‘…*it has a negative because at the moment, okay there is a time I lost my phone. It was stolen and it had that line of (****organisation name****) and it took a lot of time 3 months for them to return for me the line, so there is no data I have been checking. Because you cannot check without bundles, that’s one thing because if I check for that one that desktop in the office, that one the bundles you find that it’s not even there. Another thing since they returned for me that line, it was last month I have not been able to access any data because the password I am using and anything it doesn’t open it keeps telling me your password is wrong or your password is wrong every time every time’*. KII CHA.

b. Limited users’ experiences

Among those who failed to effectively use the system were the public health facility and some CHVs.

i. Type of facility.

Here, insufficient human resource and the perception that the application was additional work brought forth by lack of appreciation of ICT were critical issues raised. The facilities that demonstrated this were those run by the government with public resources.

“*On the other side on the man power I had one clinician who was trained that clinician stays on the other side. After seeing the clients maybe after some hours it is when she comes and opens the computer this is the computer for [Name of organisation] you see that movement something else to be done. Either one person to be assigned maybe the partner to provide the human resource it was consuming a lot of time for me the facility in fact if it one clinician she cannot manage that. Eh that’s what I can say*.” KII Health care worker.

ii. CHV attributes.

At the beginning of the interventions, CHV who had never used an e-health application, those not accustomed to using smart phone were not able to use the system adequately and these led to some drop out of CHVs. These issues presented challenges in use as demonstrated below:

“*At first it was a challenge, ‘because some of them had never used smart phones before. Okay it was excitement; it was (laughing) what do I say, excitement and mmm… anxiety at the same time. We had many when we were starting they were 11 or 13 but they dropped out coz others saw it as a challenge because others had a GPS and someone wants to report in their own house so others dropped out but those who took it they are positive.... the response is good, they are liking it* “KII CHA.

### Barriers to use

Several barriers to use of the system emerged. These were categorised into three sub-themes: (a) socio-political environment, (b) attitudes and behaviours of the users, (c) issues related to the system and (d) poor infrastructure.

- The socio-political environment

A prolonged industrial action by health workers and the contentious presidential election in the year of implementation affected the use of the technology. As depicted by the excerpt below, the industrial strike affected the use of the system as there was increased pressure and workload on those who remained and also paralysed services:

*“[…] we have everything, actually, we have all the commodities it’s only that the nurses’ strike has affected the services…... the challenge we have is only the nurses’ strike which has paralyzed these services”. KII_* Health provider

b. Attitudes and behaviour

From the observations made during implementation as well as discussion held with various key stakeholders such as health managers, healthcare worker attitudes towards the system limited use. Many development partners have in the past provided extra financial motivation to system users. Without this, the intended users preferred not to use the system. Most of the clinicians at the public facilities saw the system as additional work and not something to improve their work experiences. Unlike the private facilities that already had pre-exiting ICT/mhealth systems, the public facilities had none and this may have further affected the use of the technology. As stated above, the industrial unrest could also have negatively affected the behaviour of the health care workers. In addition, we observed that a number of users lacked basic ICT knowledge and skills which may have contributed to poor attitudes and behaviour. This was evident among the older CHVs (with the smart phones) who had challenges in navigating through the different features of the technology.

“[…] *So the health workers definitely we have issues of attitude among the health workers which you know, it change is a slow process. They cannot change overnight but it’s something that the sub-county addressed when they go to for support supervision, staffs attitude, which has a direct implication with referral system”. KII_sCHMT*

c. Issues related to the system

For some users, issues inherent to the system, including network connectivity and the phone model were major sources of concern:

*“[…] in the beginning it was very good. I could reach a lot of women more than I could in the past. When the phone has agreed to work, you could be getting out of this house and getting into this other one getting out, and then later they started the problem of hanging. You can go get somewhere and you want to, and it refuses”.* FGD CHVs

d. Poor infrastructure

In general, this being a slum setting, it is affected by the intermittent power supply and the internet coverage was weak in some of the facilities.

*“….Like right now we have been having power problems in Gikomba market from the previous fire. Now it is two weeks down the line and they haven’t gotten power coz a transformer busted something major happened ….”* KII_CHA.

The public health facilities lacked basic electric features and lack of security for gadgets resulted in the computers being kept in a store, inaccessible to a clinician.

*[…] for the clinician it was hectic in fact and most of our rooms, you see this is a store it’s not even an office it’s a store so, because of the security that is why I brought the comp here but where the clinicians sits it’s just open the door is just windows are open so security was another issue so if possible where the clinician sits its where this thing is supposed to sit so that when the client comes now the clinician can see that client my opinion.”* KII_Health provider.

### Recommendations to improve the use of the system

In many of the public health facility rooms that the clinicians used, there were no power sockets. In addition, the public facilities were not securely enforced and as a result, the desktops had to be placed in secure areas of the facilities which were different rooms from the clinician rooms. Participants suggested strategies to improve usage.

First, integrate the system with others in use and make it available on users’ regular phones.

‘*If they could send that app to our phones...we just use one phone. Either they just unlock those apps; I can also use my sim card’*. FGD CHVs.

Second, provide extra financial motivation for users as well as performance based remuneration together with using local languages in the system.

‘*Yes! But it will only solve part of the problem, the other part is how do you make them stay? So yes you can make them…you can digitize the referral tool you can digitize the coordination mechanism but how do you make them stay you must pay. So the tool needs…the tool is necessary but the tool will not succeed without additional support, yes.*’ KII Sub-County Medical Officer of Health.

Thirdly, even though the manual MoH reporting tools are in English language only. There was concern that the system being in English limited its usage by users who are not so conversant with the language. The language concerns were mainly raised by the CHVs as one CHV supervisor reiterates below;

*‘…the tool was easily adopted by the CHV’s after subsequent trials that was done but maybe further we can also improve on the language because we work with CHV’s some are very illiterate, semi illiterate and some just never went to school so when you use the system is only in English, it blocks a huge number of people who could have utilized it. So the language barrier issue should also be taken into consideration because there are those aspects that you can easily select which language you want to use whether Kiswahili or English so that everybody is accommodated*.’ KII CHA.

The health managers also recommended inclusion of a course in the basics of computer use, prior to the introduction of such innovations. It was noted that the CHVs who were very good at their daily work did not possess knowledge and skills to interact with the phone. In addition, some clinicians were not conversant with the use of computers, including powering on the device.

To facilitate adoption of the system, there was a recommendation from both the uses and health managers to provide extra motivation for users, including performance based remuneration.

## Discussion

In spite of the many pilot m-health projects implemented in Kenya, there is limited data on lessons learned especially in urban poor settings. Our study uses qualitative methods to broaden our understanding of the experiences of users of an innovative m-health solution targeted at improving decision support for CHVs, residents of some of the least developed urban slums, in Kamukunji Nairobi, Kenya. Although, not the focus of the current analysis and as reported elsewhere, implementation of the decision support system showed that the CHVs who used the application had higher knowledge of danger signs among pregnant women, new mothers and their neonates [23].

In the present analysis, overall, we found that adoption of the innovation was high at the community level compared to health facility level. The CHVs had been exposed to digital health solutions through previous interventions thus making it easier for them to embrace the innovation as it made their work easier. The mobile phone was lighter to carry and less cumbersome as compared to the paper register and it gave them a higher perceived social status in the community. The inverse was observed at the facility level, health care workers, especially at the public facilities, were of the view that the web solution gave them extra work and was cumbersome resulting from minimal or no exposure to digital interventions at the workplace.

This study highlights prevailing operational challenges and barriers to using digital health innovations in this setting [12]. Lack of basic literacy ICT skills among the health professionals was observed. To improve uptake, we offered continuous technical support, through on-job training. This was critical to increasing confidence, promoting acceptability, and utilization at the community level. Yet, due to the industrial action and infrastructural issues as well as lack of ICT readiness, on-job training did not work at health facility level.

Whereas the study highlights the potential of digitization and mobile phones as a way forward for strengthening the community health information system and decision making for community health volunteers and other health providers, it stresses some key challenges affecting implementation of digital health solutions in Kenya and related settings in SSA [4]. These include scarcity of steady power supply, lack of basic ICT skills by users, weak health systems, among others [3, 4]. Acceptance and utilization of ICT at the health facility level depended on the attitude of healthcare professionals. Poor attitudes at the health facility level were observed which may have been due to the introduction of a new and specialized concept which the health care workers were not accustomed to due to lack of skills. However, we equally observed that health workers in the setting were overwhelmed by workload – one health worker serving very many patients. Subsequently, this affected their use of the system because of the effort required to use the system for the target population, that is, the referrals from the community and continue with their normal reporting and documentation process for the rest of the population. This may have worked better if the workload of health care workers was distributed among patients and if the system was used for all the patients. The workload was exacerbated by the prolonged industrial strike that left a thin workforce in the health facilities as a result of underlying and long-term frustrations of health care workers especially in the public health sector [24]. This not only affected the use of the system, but delivery and utilization of healthcare services [25] This finding reiterates the WHO’s call to invest in strengthening health systems before investing in digital health solutions as these too rely on functioning health systems [5]. Kenya, like other countries in sub-Saharan Africa, needs a healthcare workforce that is not only motivated but empowered with skills sets that meet the current epidemiological and demographic needs of its population. Inasmuch as infrastructure, vaccines, medicines and technologies are important; investing billions of dollars in health technologies, commodities and facilities is counterproductive, to improving population health if there are no skilled, motivated and adequate personnel to operate and deliver the services.

As Kenya and other countries in the SSA region progress to ICT revolution in health, there is an urgent need to ensure that the basic support is met. Lack of ICT equipment, sporadic electricity supply and lack of power outlets in the public facility coupled with insecurity in the environs were major hurdles faced by the intervention. Failure to create an enabling environment will always be an impediment to the successful implementation of such interventions. Some of the users experienced mobile network interruptions. As such, engaging mobile service providers and have them on board to address infrastructure sustainability is critical to successful implementation. Heavy investments are required in equipment acquisition and maintenance as well as internet solutions.

Among the barriers to effective utilisation of the system was the socio-political climate at the end of 2016 and in 2017 which adversely affected the utilization of the system at the health facility level. A long electioneering period in the country characterised by uncertainty and bouts of insecurity as well as a nationwide health workers’ strike affected the full implementation of the activities.

During the implementation, it was noted that weak support for the health systems in general and for the community health strategy in particular, continues to affect implementation of healthcare services in Kenya [26]. This also had an impact on the activities implemented. This observation is in agreement with existing reports that despite being clearly defined on paper and its growing role in delivering vital primary health care services, there is limited government support [27, 28]. Several users expected extra financial motivation while others could not comfortably use the phones and desktop computers provided. In this light, the study further stresses the necessity of considering the behavioural determinants of data collection activities in the strengthening of health information systems. There is also need to explore sustainable models to motivate users to utilize the system and not just financial motivation.

On the recommendations, there were calls for integration of new solutions with existing ones. The CHVs were using the provided handset, strictly for work and this proved challenging as they had to carry their personal phones as well as the work one. On the other hand, the health facilities have other programmatic systems for electronic medical records such as HIV/AIDS, Tuberculosis that are supported by different partners. With the introduction of mPAMANECH, users had to switch from one system to another. As the country embraces digital solutions, there is a need to debate on whether proposed systems should interoperate; the ability to exchange data between two or more systems) or integrate; joining distinct systems into one to facilitate smooth implementation, operation and efficient use these technologies. The EAC health secretariat has recommended that the EAC Partner States develop common platforms to facilitate interoperability. This is necessary to promote learning from best practices.

## Study limitations

While our study provides a rich context to m-health implementation in this setting (in the Nairobi slums), we only elicit the perspectives of individuals resident and/or working in this community. This may limit our application of our theory of change, and ability to make recommendations for digital and public health interventions. Furthermore, the short implementation period needs to be taken into account, especially in relation to the socio-political environment that prevailed during the period.

## Conclusions

The study demonstrates the feasibility and acceptability of this type of research in a previously under-researched sub-population. It serves as a basis for future work that could highlight opportunities to respond to the persistent challenges surrounding m-health implementation in low resource settings. Before countries move towards the use of digital technologies in health, the level of preparedness should be determined in order to save on time and money. Lastly, as more healthcare delivery models are developed, harnessing the potential of digital technologies, strengthening health systems is critical as this provides the backbone on which such innovations draw support.

Based on the lack of ICT skills observed, we recommend that the government and its partners invest in empowerment of in-service health workers through capacity building on ICT and continuous technical support. This may include integrating e-health into existing curricula, continuous professional training and promote the use of distance learning for continuous education. In this way, the health professionals will acknowledge and appreciate the value of e-health in strengthening the health system.

In the development and deployment of digital health solutions, continuous support is required at all levels from the development of user-friendly and easy to use the application to implementation. Where possible, development should involve the users from the development of the concept and be flexible to the changing needs of the users.

Subnational health departments, with assistance from the national government, need to explore modalities to operationalize and enforce the e-health policy and strategies to create and sustain an enabling environment for ICT in health. As one of the major challenges of rolling out e-health solutions is lack of commitment from the county governments whose mandate is to implement already existing policies, there is a need to develop solutions. In addition, the County health department ought to strengthen coordination and joint efforts to achieve greater impact by involving all stakeholders to avoid overburdening the users and duplication of efforts.

In addition, adoption of ICT is a complex process and will require strategic partnerships. There is need to strengthen public-private partnerships and inter-ministerial engagement to ensure that the pillars in ICT in health are adequately supported for successful implementation and efficient utilization of investments. Joint efforts involving academia, industry, policymakers and practitioners are necessary to drive digital health technology agendas within countries.

Lastly, there is a need to invest in research to promote evidence-based solutions and decision making at all levels and develop solutions in the contexts that they apply.

## Supplementary information

**Additional file 1.** Study guide.

## Data Availability

The datasets generated and/or analysed during the current study are not publicly available because of the organisation’s guidelines on data sharing and access which state that data is made available to the public domain after 24 months on the APHRC Microdata portal: http://microdataportal.aphrc.org/index.php/catalog. In the meantime, the data can be availed from the principle investigator, Pauline Bakibinga; email pbakibinga@aphrc.org, upon reasonable request.

## Abbreviations

AMREF ESRC: Amref Health Africa’s Ethics and Scientific Research Committee
App: Application
CHA: Community health assistant
CHV: Community health volunteer
CPD: Continuous professional development
CU: Community Unit
DST: Decision support tool
EAC: East African Community
E-Health: Electronic health
FGD: Focus group discussions
ICT: Information and Communications Technology
IDI: In-depth interview
KII: Key informant interview
M-Health: Mobile health
MNH: Maternal and Newborn Health
MoH: Ministry of Health
mPAMANECH: Mobile Partnership for Maternal, Newborn and Child Health
sCHMT: Sub-County Health Management Team
SSA: Sub-Saharan Africa
WHO: World Health Organisation
