# Supplementary material for: Challenges and prospects for implementation of community health volunteers’ digital health solutions in Kenya: a qualitative study
Source: BMC Health Serv Res. 2020 Sep 21;20:888. doi: 10.1186/s12913-020-05711-7 (PMC7507673; doi:10.1186/s12913-020-05711-7)
Supplement: Supplementary file 1 — Additional file 1. Study guide. [file 12913_2020_5711_MOESM1_ESM.pdf]

Using a decision-support smartphone application to enhance Community Health Volunteers' effectiveness in reducing maternal complications and reducing neonatal deaths in the informal settlements of Nairobi, Kenya

Thematic Interview Guide

I. Focus Group Discussion (FGD) Guide

Community Health Volunteers (CHVs)

ICE BREAKER

- Kindly explain how one becomes a community health volunteer? Please elaborate.

Section A: Experience working in the community

Q1. Tell me about your experience working in the community as a Community Health Volunteer, over the last one year?

Probes

a) Have you received any training on how to identify danger signs?
b) What do you use to identify danger signs in newborns, pregnant and postpartum mothers? How easy are these methods?
c) How do you handle neonatal complications? How about the maternal complications i.e. among pregnant women (including during deliveries) and postpartum women?
d) At what point do you refer to complicated cases?
e) What challenges do you face while executing your duties as a CHV?
f) What do you think can improve your experience as a CHV?
g) In your own view, what do you know about the relationship between the CHVs and the community they serve? Elaborate (negative or positive)?
h) How can this relationship be strengthened?
i) In your view, what was the response of the community/households to you using the phone for data collection?

Section B: Experience working with health facilities and referral mechanism

Q1. Tell me about your experience working with the health facilities in the area over the past one year? With you using the phone have there been any changes? Please elaborate.

Probes

a) How have you linked with health facilities?
b) In your opinion were the patients satisfied with the services?
c) What are the perceived perceptions about the **quality**, **accessibility** and **affordability** regarding:
    i. Services offered in the facilities
    ii. The existing FP and maternal health services?
    iii. Effectiveness of referral system
    iv. Responsiveness of the services offered to the needs of the community?
    v. Barriers to utilization of the health services.
d) Challenges and Opportunities (how to assist mothers and children to access care), improve their work lives (reporting, knowledge and skills)
e) In your own view, do you think the relationship between the CHVs and the health facilities has changed? Elaborate (negative or positive)
f) How can this relationship be strengthened?

Q2. Tell me how the referral mechanism from the community to the health facility works?

Probes

a) What is the role of CHVs in referrals? Please elaborate?
b) Which health facilities do you usually refer to your clients to? Are these public or private facilities? Why do you prefer these facilities?
c) How do you follow up patients referred to ensure they go to the health facilities?

d) How do you get feedback from the facilities on referrals done?
e) What challenges are in the referral system?
f) What do you think can be done to strengthen the referral system?

Section C: Community Health Information Systems

Q1. I would like you to talk about the information collection/reporting systems/tools that you have used over the past one year

Probes

a) Generally, how do you feel about the information collection/reporting system currently in use?
b) How do you collect information from the households allocated?
c) What do you use to collect information from the households allocated?
d) On average, how much time does it take to collect information from one household?
e) How do you report information collected from households?
f) How often is your work reviewed? Who reviews your work?
g) What are the advantages and disadvantages/challenges of the current system?
h) How in your opinion do you think the current system of information collection and reporting can be improved?
i) If you have heard of any health related mobile applications used in this community, what is your opinion?

Q2. Now I would like us to talk about your opinions about household information collection activities as part of your work as a CHV.

Probes

a) What are the reasons you collect information from households?
b) As a CHV, why should you collect information from households? Why shouldn't you collect information from households?
c) Do you think it is important that you collect information from households? Elaborate.
d) Do you feel respected or disrespected by the community while doing information collection activities? Why?
e) Do you think that the community trusts you with the information you collect from them? Why?

Q3. To extend our discussion I would like us to talk about the use of information collected by CHVs.

Probes

a) How do you think the information collected by you is used? Elaborate
b) By whom is the information you collect used?
c) Who should use the information collected by you?
d) How have you seen the information collected by you is used in the community? Elaborate
e) How have you used the information that you have collected from households? Elaborate and give an example.

Q4. In your opinion, does the information that you collect from households improve the health situation in your community? Elaborate

Probes

a) Have you seen that the information collected by you has influenced the decision-making or action planning at the household level? Community level?
b) Who makes decisions about activities to improve health in the community?
c) What influences decisions made about health in your community?

Q5. Thank you for the views you have shared. So far; I would like to talk about how the information you collect from households is given back to the community. Through what ways has information collected by you been given back to the community?

Probes

a) Have you seen the community chalkboard? What does the information displayed on the chalkboard mean?
b) Do you know about community health committees? Is there a functional community health committee in this community? Do you know about their roles (list them)
c) Have you attended a community health dialogue day? How do you get to know about community dialogue days?
d) What happens on a community dialogue day? Who facilitates community dialogue days?
e) Have you attended a community health action day? How do you get to know about community action days?
f) What happens on a community health action day? Who facilitates community health action days?
g) Do you think there are better ways for you to relay the information you collect back to the community?

<u>Section D: Experience with m-PAMANECH</u>

Q6. Tell me about your experience working with the phone

Probes

a) What are your views on the use of a decision support tool for CHVs to collect/report information & make referrals/ follow-ups?
b) What was the impact of using the phone on your work in the community? Elaborate (positive or negative)
c) Overall, what do you think about the user-friendliness of this product?
d) Have you faced any challenges functioning of the application? Elaborate.
e) What do you think can be done to address these challenges?

II. In- depth Interview Guide (IDI) - ***Community Health Volunteers (CHVs)***

Section A: CHV Experiences

Q1. Tell me about your experience working in the community as a Community Health Volunteer, over the last one year?

Probes

a) Have you received any training on how to identify danger signs?
b) Have you received training on electronic mobile reporting?
c) How are you applying these systems?
d) What do you use to identify danger signs in newborns, pregnant and postpartum mothers? How easy are these methods?
e) How do you handle neonatal complications? How about the maternal complications i.e. among pregnant women (including during deliveries) and postpartum women?
f) At what point do you refer to complicated cases?
g) What challenges do you face while executing your duties as a CHV?
h) What do you think can improve your experience as a CHV?
i) In your own view, what do you know about the relationship between the CHVs and the community they serve? Elaborate (negative or positive)
j) How can this relationship be strengthened?
k) In your view, what was the response of the community/households to you using the phone for data collection?

Section B: Experience with m-PAMANECH

Q2. What was your experience using the phone?

Probes

a) What was the impact of using the phone on your work in the community? Elaborate (positive or negative)
b) Do you think that you would like to use this mobile app frequently?
c) What do you think about the functionality of the mobile app:
   i. Unnecessarily complex?
   ii. Easy to use?
   iii. I would need assistance to be able to use this mobile app
   iv. Various functions in this mobile app were well integrated
   v. Inconsistency of the app
   vi. I would imagine that most people would learn to use this mobile app very quickly
   vii. Confidence using the app
   viii. Very cumbersome/awkward to use
   ix. I needed to learn a lot of things before I could get going with this mobile app

III. Key Informant Interview Guide (KII)

a. *Community Health Assistant (CHA)*

ICE BREAKER

- Kindly tell me about how one becomes a CHA?

Section A: Experience working as a CHA in the community

Q1. Tell me about your roles as a CHA in provision of health services to people in this community

Probes

a) Health status of the community
b) Interaction with the CHVs
c) Experiences working with the CHVs

Q2. Tell me about your experience working in the community as a Community Health Assistant, over the last one year?

Probes

a) Have you received any training on electronic mobile reporting?
b) How are you applying these systems?
c) What challenges do you face while executing your duties as a CHA?
d) What do you think can improve your experience as a CHA?
e) In your own view, what do you know about the relationship between the CHAs and the community you serve? Elaborate (negative or positive)
f) How can this relationship be strengthened?
g) In your view, what was the response of the community/households using the paper-based tools (MOH 514 book) for data collection?
h) In your view, what was the response of the CHVs using the paper-based tools (MOH 514 book) for data collection?

Section B: Experience working with health facilities and referral mechanism

Q1. Over the past one year, tell me about your experience working with the health facilities in the area? With you using the web portal have there been any changes? Please elaborate.

Probes

a) How have you linked with health facilities?
b) In your opinion were the patients satisfied with the services?
c) What are the perceived perceptions about the **quality**, **accessibility** and **affordability** regarding:
   vi. Services offered in the facilities
   vii. The existing FP and maternal health services?
   viii. Effectiveness of referral system
   ix. Responsiveness of the services offered to the needs of the community?
   x. Barriers to utilization of the health services.
d) Challenges and Opportunities (how to assist mothers and children to access care), improve their work lives (reporting, knowledge and skills)
e) In your own view, do you think the relationship between the CHVs and the health facilities has changed? Elaborate (negative or positive)
f) How can this relationship be strengthened?
g) In your own view, do you think the relationship between the CHAs and the health facilities has changed? Elaborate (negative or positive)

h) How can this relationship be strengthened?

Q2. Tell me about the referral mechanism from the community to the health facility, over the past one year?

Probes

a) What is the role of CHVs in referrals? Please elaborate?
b) What is the role of CHAs in referrals? Please elaborate?
c) Which health facilities do CHVs usually refer clients to? Are these public or private facilities? Why do they prefer these facilities?
d) How do CHVs follow up patients referred to ensure they go to the health facilities?
e) How do CHVs get feedback from the facilities on referrals done?
f) What challenges are in the referral system?
g) What do you think can be done to strengthen the referral system?

Section C: Community Health Information Systems

Q1. I would like you to talk about the information collection/reporting systems/tools that CHVs and health facilities use

Probes

a) Generally, how do you feel about the information collection/reporting system currently in use?
b) How do CHVs collect information from the households allocated?
c) What do CHVs use to collect information from the households allocated?
d) On average, how much time does it take to collect information from one household?
e) How do you report information collected from households?
f) Do you receive any information from the health facilities? How do you use it?
g) How often is your work reviewed? Who reviews your work?
h) How often do you review the CHVs work?
i) What are the advantages and disadvantages/challenges of the current system?
j) In your opinion, how do you think the current system of information collection and reporting can be improved?

Q2. Now I would like us to talk about your opinions on the household information collection activities as part of your work as a CHA

Probes

a) In your opinion, what are the reasons CHVs collect information from households?
b) As a CHA, why should you collect information from CHVs? Why shouldn't CHAs collect information from CHVs?
c) Do you think it is important that you collect information from CHVs? Elaborate
d) Do you feel respected by the community while doing information collection activities? Why? Do you feel disrespected by the community while doing information collection activities?
e) Do you think that the community trusts you with the information you collect from them? Why?

Q3. Now I would like us to talk about the use of the information collected by CHVs.

Probes

a) How do you think the information collected by you is used? Elaborate
b) By whom is the information you collect used?
c) Who should use the information collected by you?
d) How have you seen the information collected by you is used in the community? Elaborate.

e) How have you used the information that you have collected from households? Elaborate and give an example.

Q4. In your opinion, does the information that you collect from households improve the health situation in your community? Elaborate

Probes

a) Have you seen that the information collected by you has influenced the decision-making or action planning at the household level? Community level?
b) Who makes decisions about activities to improve health in the community?
c) What influences decisions made about health in your community?

Q5. Now I would like to talk about how the information you collect from households is given back to the community. Through what ways has information collected by you been given back to the community?

Probes

a) What does the information on the chalkboard mean?
b) Is there a functional community health committee in this community? Who are they? What are their roles? (list them)
c) How often do you organize community health dialogue days? What happens on a community dialogue day? Who facilitates community dialogue days?
d) How often do you participate in community health action days?
e) What happens on a community health action day? Who facilitates community health action days?
f) Do you think there are better ways for you to relay the information you collect back to the community?

Section D: Experience with m-PAMANECH

Q6. Tell me about your experience working with the web portal?
Probes

a) Overall, what do you think about the user-friendliness of this product?
b) What was the impact of **you** using the web portal on your work in the community? Elaborate (positive or negative)
c) Have you faced any challenges functioning of the application? Elaborate.
d) What do you think can be done to address these challenges?
e) What are your views on the use of a decision support tool for CHVs to collect/report information & make referrals/ follow-ups?
f) In your view, what was the response of the CHVs on using phones for data collection?

Q6. What are your views on the use of the decision support tool for CHVs to collect/report information & make referrals/ follow-ups?

b. Health Providers

ICE BREAKER

- Kindly tell me about your position or designation in this facility?
- How long have you served in this position?

<u>Section A: Health care services and community referrals</u>

Q1. Tell me about your roles as a health provider in provision of health services to people in this community

Probes

a) Health status of the community
b) Interaction with the CHVs
c) Interaction with sCHMTs

Q2. Describe to me the catchment area of this facility? What kind of health services are offered to the community it serves? What services are mostly utilized?

Probes

a. In your opinion, are the services offered responsive to the needs of the community? Are the services offered of quality? Affordable? Elaborate for each response.
b. In your opinion, do you think the facility is accessible? Tell me more.
c. What can be done to make things better in these health facilities to better serve the community? Tell me more.

Q3. Over the past one year, describe to me how the link between this health facility and community units in this area have been created and operated.

Probes

a. What are your views on the referral system by CHVs? How do the CHVs do the referrals to this facility?
b. How do you give feedback to the CHV? Elaborate for each response
c. What are the challenges in the community referral system? What can be done to strengthen it?
d. In your own view, do you think the relationship between the CHVs and this health facility is negative or Positive? Elaborate
e. What can be done to strengthen that relationship?

<u>Section B: Health Information Management Systems</u>

Q1. I would like you to talk about the information collection/reporting systems/tools that are used in this health facility

Probes

a) Generally, how do you feel about the information collection/reporting system currently in use?
b) How does this health facility collect information about the community from the CHVs?
c) How does this health facility receive information about the community from the CHVs?
d) How do you report information collected from your facility?
e) On average, how much time does it take to summarize and report these data?
f) How often is your work reviewed? Who reviews your work?
g) What are the advantages and disadvantages/challenges of the current system?
h) In your opinion, how do you think the current system of information collection and reporting can be improved?

Q2. **Tell me about the reporting tools used for data collection/reporting by CHVs.**

Probe:

a) What tools are used to collect information from the community linked to this facility? Are they paper or electronic paper?
b) Generally, how do you feel about the data collection /reporting system currently in use? What are the advantages and disadvantages/challenges of the current system?
c) What is your opinion on a mobile application for information collection and reporting?

Q2. Now I would like us to talk about your opinions on the household information collection activities as part of your work as a health provider

Probes

a) In your opinion, what are the reasons you households collect information in this facility?
b) As a health provider, why should you collect household information? Why shouldn't you?
c) Do you think it is important that you receive household information from CHVs? Elaborate
d) Do you think that the community trusts you with the information you collect from them? Why?

Q3. Now I would like us to talk about the uses of information that is collected at this facility.

Probes

a) How do you think the information collected by you is used? Elaborate
b) By whom is the information you collect used?
c) Who should use the information collected by you?
d) How is the information that you have collected from your patients used in the community? Elaborate and give an example.

Q4. In your opinion, does the information that you collect from patients improve the health situation in the community? Elaborate

Probes

a) How have you seen the information collected by you influencing decision-making or action planning?
b) Who makes decisions about activities to improve health in the community?
c) What influences decisions made about the health of the communities that this health facility serves?

<u>Section C: Experience with m-PAMANECH</u>

Q5. Tell me about your experience working with the web portal?

Probes

a) What are your views on the use of the web portal to collect/report information & make referrals/ follow-ups?
b) What was the impact of the CHAs using the web portal on your work in the community? Elaborate (positive or negative)
c) Overall, what do you think about the user-friendliness of this product?
d) Have you faced any challenges functioning on the portal? Elaborate.
e) What do you think can be done to address these challenges?

Q6. What are your views on the use of the decision support tool for CHVs to collect/report information & make referrals/ follow-ups?

c. KII: *Sub County focal person*

ICE BREAKER

- Kindly tell me about your position or designation in this sub county?
- How long have you served in this position?

Section A: Roles of Sub County focal person

Q1. Tell me about your roles as the sub county focal person in provision of health services to people in this community?

Probes

a) Health status of the community
b) Interaction with the CHVs
c) Interaction with sCMOH

Q2. Over the past one year, what has your experience been working in the community as the Sub County focal person?

Probes

a) Have you received any training on electronic mobile reporting?
b) How are you applying these systems?
c) What challenges do you face while executing your duties as a Sub County focal person?
d) What do you think can improve your experience as the Sub County focal person?
e) In your own view, what do you know about the relationship between the CHAs/CHVs and the community you serve? Elaborate (negative or positive)
f) How can this relationship be strengthened?
g) In your view, what was the response of the community/households using the paper-based tools (MOH 514 book) for data collection?
h) In your view, what was the response of the CHVs using the paper-based tools (MOH 514 book) for data collection?

Section B: Experience working with health facilities and referral mechanism

Q1. Tell me about your experience working with the health facilities in the area in the past one year? With you using the web portal have there been any changes? Please elaborate.

Probes

a) How have you linked with health facilities?
b) In your opinion were the patients satisfied with the services?
c) What are the perceived perceptions about the **quality**, **accessibility** and **affordability** regarding:
    xi. Services offered in the facilities
    xii. The existing FP and maternal health services?
    xiii. Effectiveness of referral system
    xiv. Responsiveness of the services offered to the needs of the community?
    xv. Barriers to utilization of the health services.
d) Challenges and Opportunities (how to assist mothers and children to access care), improve their work lives (reporting, knowledge and skills)
e) In your own view, do you think the relationship between the CHVs and the health facilities has changed? Elaborate (negative or positive)
f) How can this relationship be strengthened?
g) In your own view, do you think the relationship between the CHAs and the health facilities has changed? Elaborate (negative or positive)
h) How can this relationship be strengthened?

Q2. Tell me how the referral mechanism from the community to the health facility has been working over the past one year?

Probes

a) What is the role of CHVs in referrals? Please elaborate?
b) What is the role of CHAs in referrals? Please elaborate?
c) Which health facilities do CHVs usually refer clients to? Are these public or private facilities? Why do they prefer these facilities?
d) How do CHVS follow up patients referred to ensure they go to the health facilities?
e) How do CHVs get feedback from the facilities on referrals done?
f) What challenges are in the referral system?
g) What do you think can be done to strengthen the referral system?

Section C: Community Health Information Systems

Q1. I would like you to talk about the information collection/reporting systems/tools that CHVs and health facilities use

Probes

a) Generally, how do you feel about the information collection/reporting system currently in use?
b) How do CHVs collect information from the households allocated?
c) What do CHVs use to collect information from the households allocated?
d) On average, how much time does it take to collect information from one household?
e) How do you report information collected from households?
f) Do you receive any information for the health facilities? How do you use it?
g) How often is your work reviewed? Who reviews your work?
h) What are the advantages and disadvantages/challenges of the current system?
i) How in your opinion do you think the current system of information collection and reporting can be improved?
j) What is your opinion about mobile applications for data collection and reporting?

Q2. In your opinion do you think the CHV decision support tool has made CHVs perform duties and responsibilities effectively? How is that?

Probe

a) What were your expectations of the project?
b) Have they been met? (elaborate)
c) **What could have been done better? (elaborate)**

Q3. Now I would like us to talk about your opinions on the household information collection activities as part of your work as the sub county focal person

Probes

a) In your opinion, what are the reasons CHVs collect information from households?
b) As the sub county focal person, why should information be collected by CHVs? Why shouldn't information be collected by CHVs?
c) Do you think that the community trusts you with the information you collect from them? Why?

Q4. I would like us to talk about the use of information collected by CHVs.

Probes

a) How do you think the information collected by you is used? Elaborate
b) By whom is the information you collect used?
c) Who should use the information collected by you?
d) How have you seen the information collected by you is used in the community? Elaborate

e) How have you used the information that you have collected from households? Elaborate and give an example.

Q5 In your opinion, does the information that CHVs collect from households improve the health situation in the sub county? Elaborate

Probes

a) How have you seen the information collected influencing decision-making or action planning?
b) Who makes decisions about activities to improve health in this sub county?
c) What influences decisions made about health in this sub county?

Q6. Now I would like to talk about how the information collected from households is given back to the community. Through what ways has information collected by CHVs been given back to the community?

Probes

a) What does the information on the chalkboard mean?
b) Is there a functional community health committee in this community? Who sits in that committee? What are their roles? (list them)
c) How often do you organize community health dialogue days? What happens on a community dialogue day? Who facilitates community dialogue days?
d) How often do you participate in community health action days?
e) What happens on a community health action day? Who facilitates community health action days?
f) Do you think there are better ways to relay the information collected back to the community?

Section D: Experience with m-PAMANECH

Q7. Tell me about your experience working with the web portal?

Probes

f) What are your views on the use of the web portal to collect/report information & make referrals/ follow-ups?
g) What was the impact of the CHAs using the web portal on your work in the community? Elaborate (positive or negative)
h) Overall, what do you think about the user-friendliness of this product?
i) Have you faced any challenges functioning on the portal? Elaborate.
j) What do you think can be done to address these challenges?

Q8. What are your views on the use of the decision support tool for CHVs to collect/report information & make referrals/ follow-ups?

d. KII: *SUB-COUNTY MEDICAL OFFICER OF HEALTH (SCMOH*)

ICE BREAKER

Section A: Role as SCMOH

Q1. Tell me about your role as the SCMOH in the provision of health services in this community? Please elaborate

Probes

a) Support supervision, sub county meetings & stakeholder forums

Section B: Healthcare services

Q1. How is it working with health facilities?

Probes

a) Reporting?

b) Compliance with regulations

c) Perceived quality, accessibility and affordability regarding the existing FP and maternal health services, effectiveness of referral system, and barriers to utilization of the health services.

d) Challenges and Opportunities (how to assist mothers and children to access care), reporting, compliance with regulations by health facilities

<u>Section C: Engagement with CHVs and Health Information Systems</u>

Q1. Tell me about your experience working with CHVs

Probes:

a) How do you engage with CHVs?

b) Their effectiveness in the community?

<u>Section D: Experience with m-PAMANECH</u>

Q2. In your opinion do you think a CHV decision support tool would make CHV perform duties and responsibilities effectively? How is that?

Probe

a) What were your expectations of the project?

b) Have they been met? (elaborate)

c) **What could have been done better? (elaborate)**
